# Supplementary material for: Case Series of 11 CDH1 Families (47 Carriers) Including Incidental Findings, Signet Ring Cell Colon Cancer and Review of the Literature
Source: Genes (Basel). 2023 Aug 25;14(9):1677. doi: 10.3390/genes14091677 (PMC10530895; doi:10.3390/genes14091677)
Supplement: Supplementary file 1 [file genes-14-01677-s001.zip › genes-2531128-supplementary.pdf]

Supplementary Materials

Family 1 :

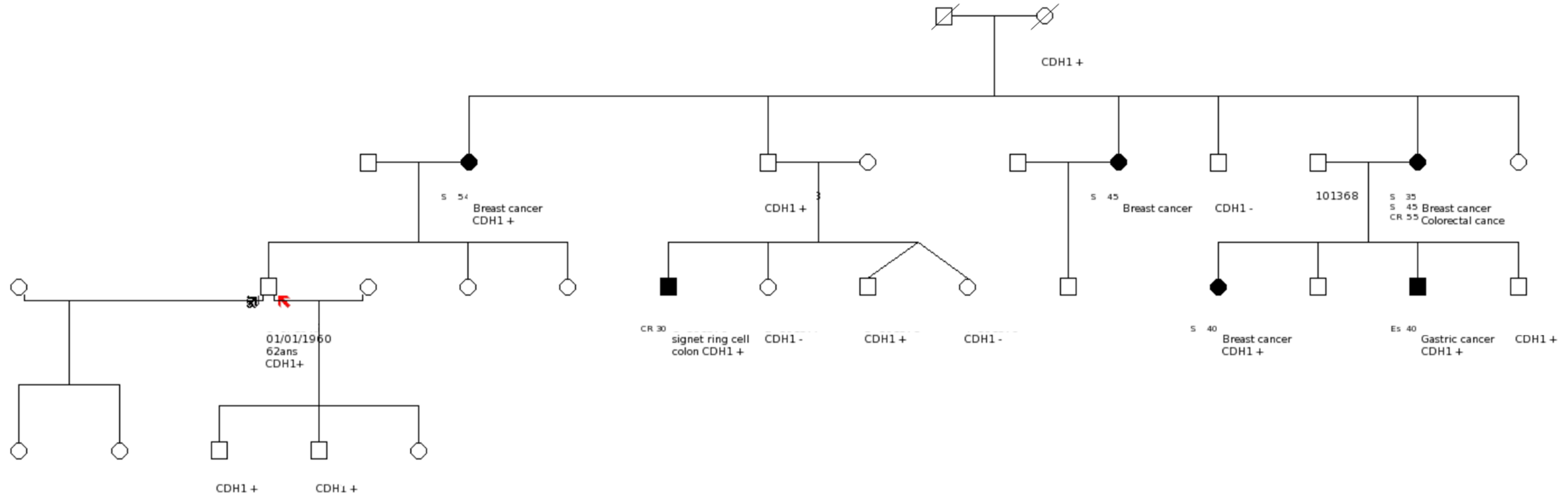

S : Breast cancer

Pr : Prostate cancer

ES : gastric cancer

LBC : lobular breast cancer

PM : lung cancer

CR : colorectal cancer

Family 2 :

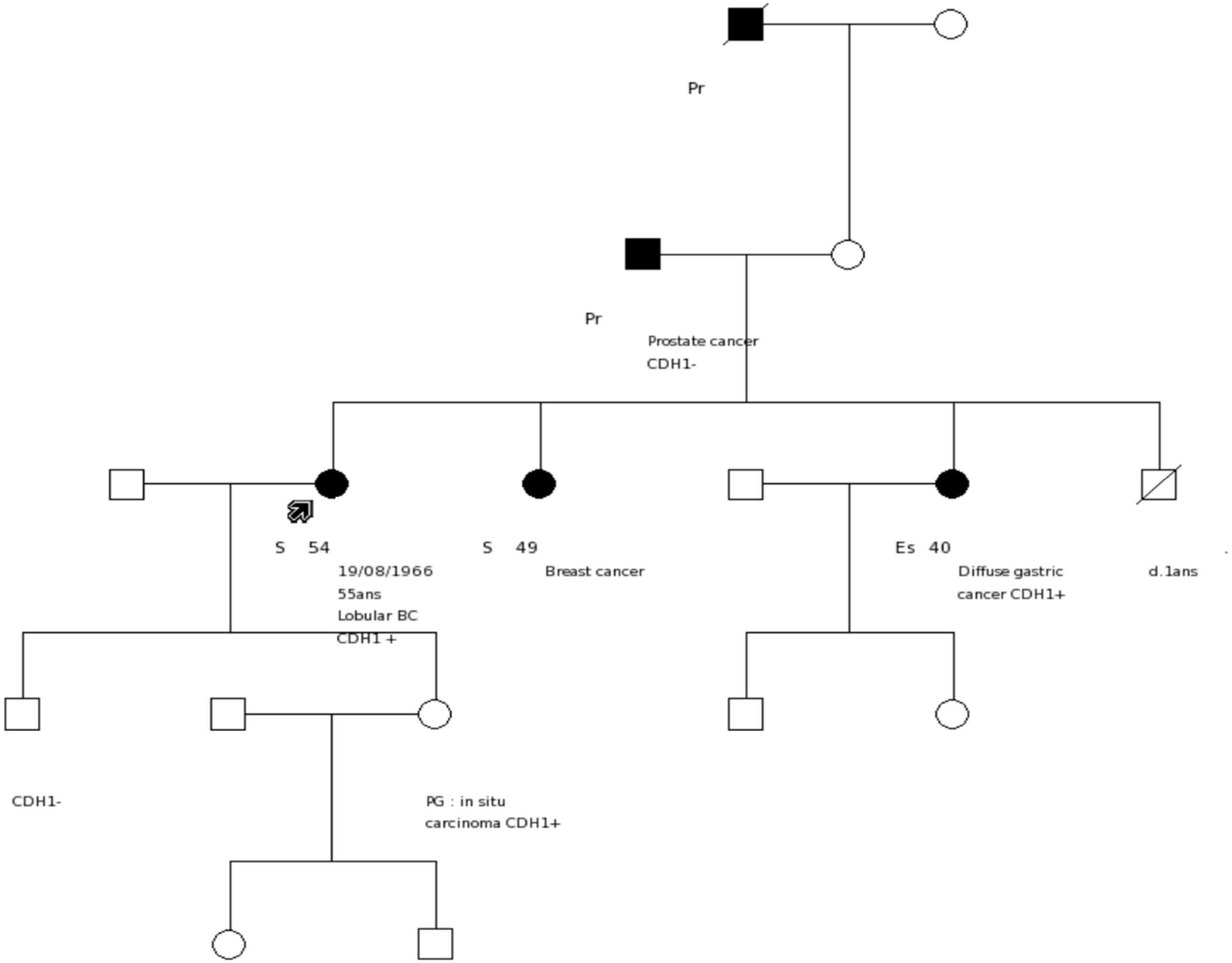
Pr
Pr
Prostate cancer
CDH1-
S 54
19/08/1966
55ans
Lobular BC
CDH1 +
S 49
Breast cancer
Es 40
Diffuse gastric
cancer CDH1+
d.1ans
CDH1-
PG : in situ
carcinoma CDH1+

Family 3 :

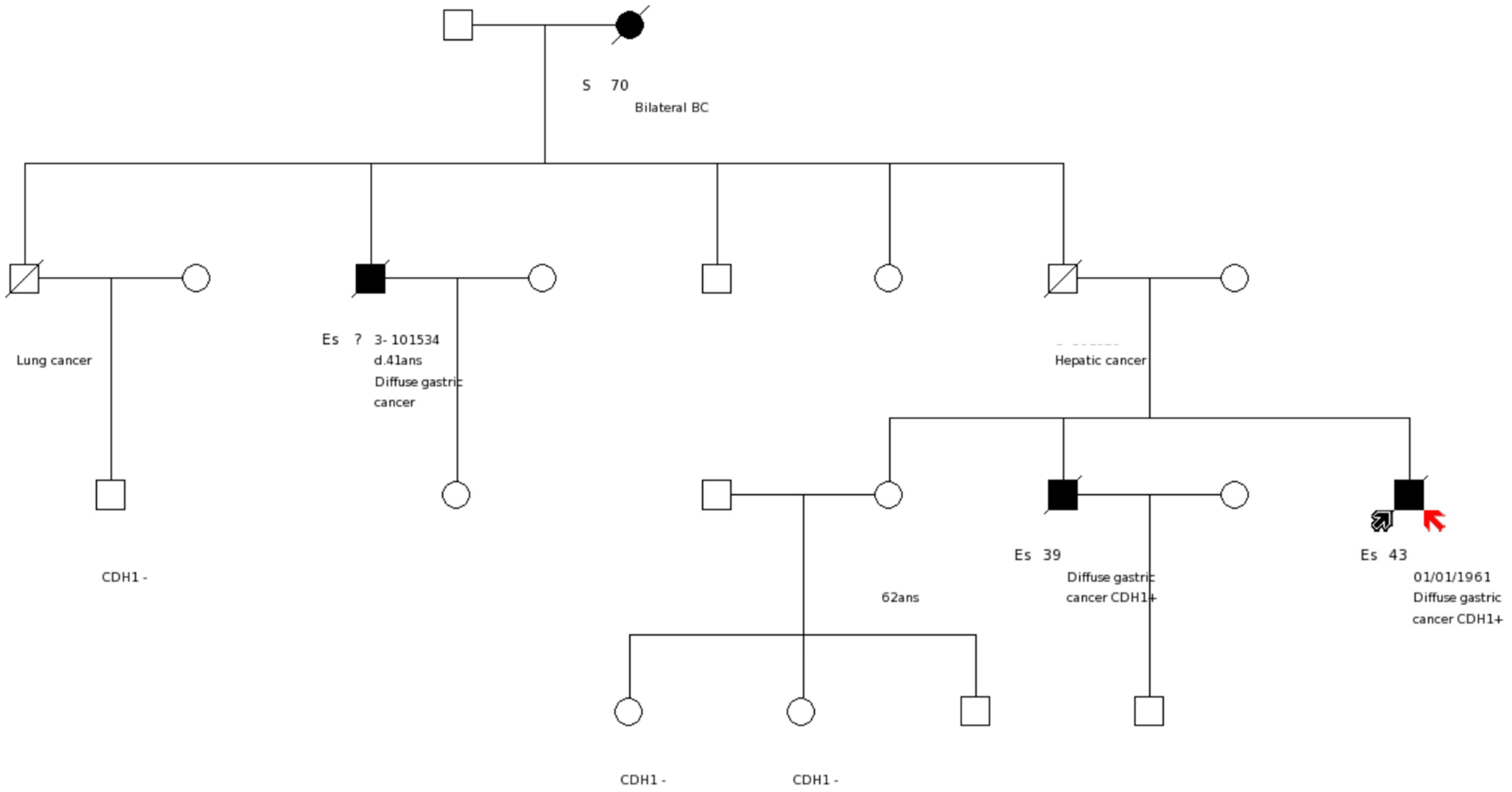
S 70
Bilateral BC
Lung cancer
Es ? 3- 101534
d.41ans
Diffuse gastric
cancer
Hepatic cancer
CDH1 -
62ans
Es 39
Diffuse gastric
cancer CDH1+
Es 43
01/01/1961
Diffuse gastric
cancer CDH1+
CDH1 -
CDH1 -

Family 4 :

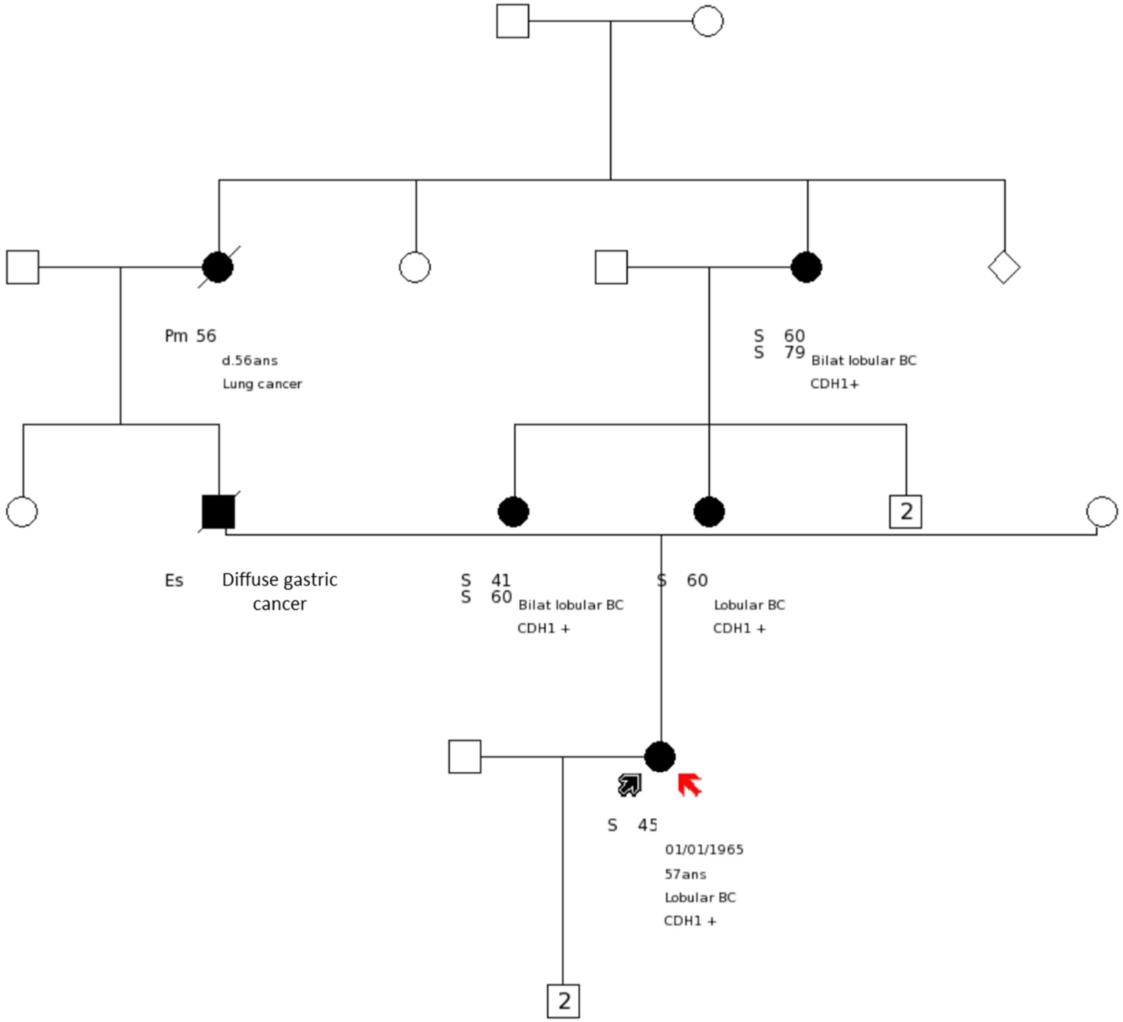

Pm 56
d.56ans
Lung cancer
S 60
S 79
Bilat lobular BC
CDH1+
2
Es
Diffuse gastric cancer
S 41
S 60
Bilat lobular BC
CDH1 +
S 60
Lobular BC
CDH1 +
S 45
01/01/1965
57ans
Lobular BC
CDH1 +
2

Family 5 :

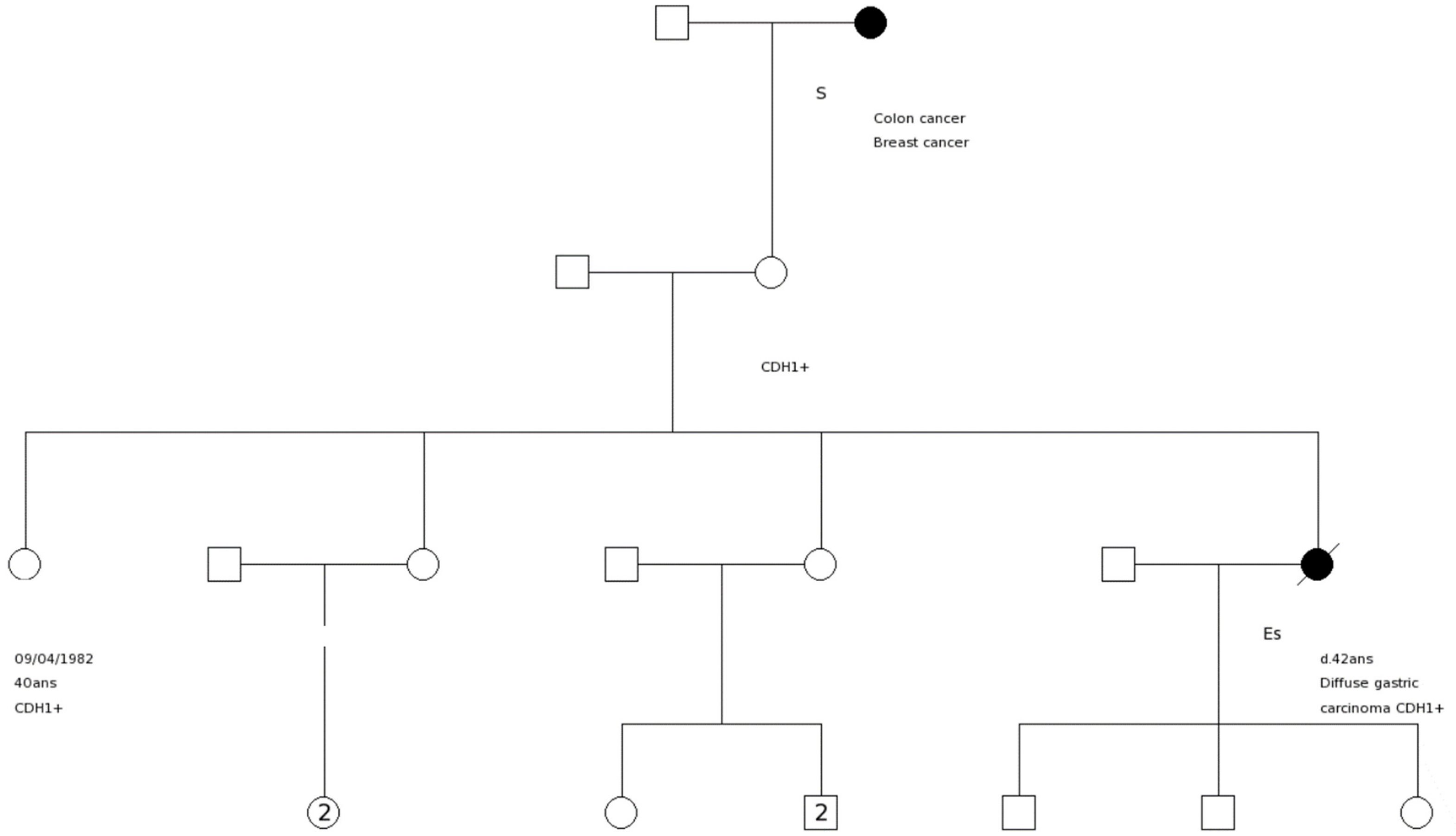
S
Colon cancer
Breast cancer
CDH1+
09/04/1982
40ans
CDH1+
2
2
Es
d.42ans
Diffuse gastric
carcinoma CDH1+

Family 6 :

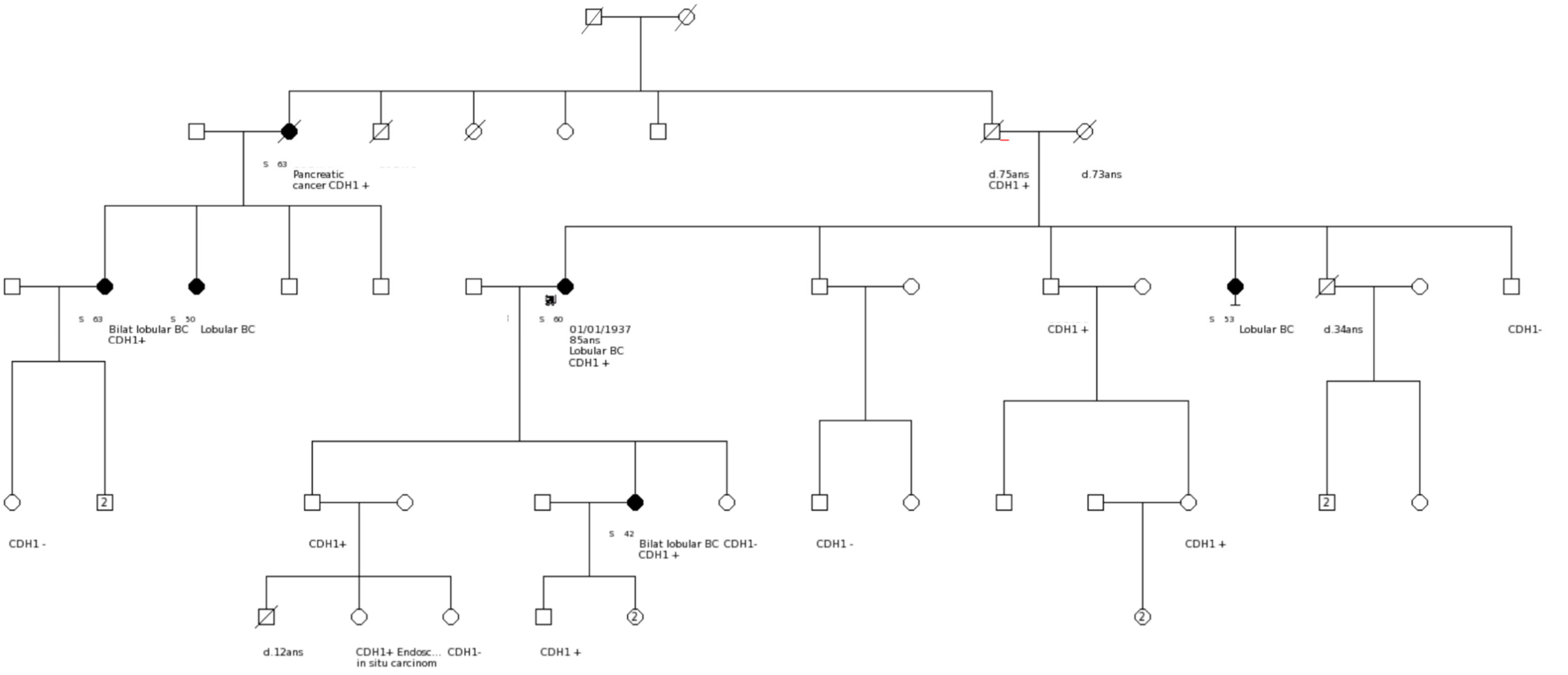

S 63
Pancreatic cancer CDH1 +
d.75ans CDH1 +
d.73ans
S 63
Bilat lobular BC CDH1+
S 50
Lobular BC
S 60
01/01/1937 85ans Lobular BC CDH1 +
CDH1 +
S 53
Lobular BC
d.34ans
CDH1-
2
CDH1 -
CDH1+
S 42
Bilat lobular BC CDH1 +
CDH1-
CDH1 -
CDH1 +
2
d.12ans
CDH1+ Endosc... in situ carcinom
CDH1-
CDH1 +
2
2

Family 7 :

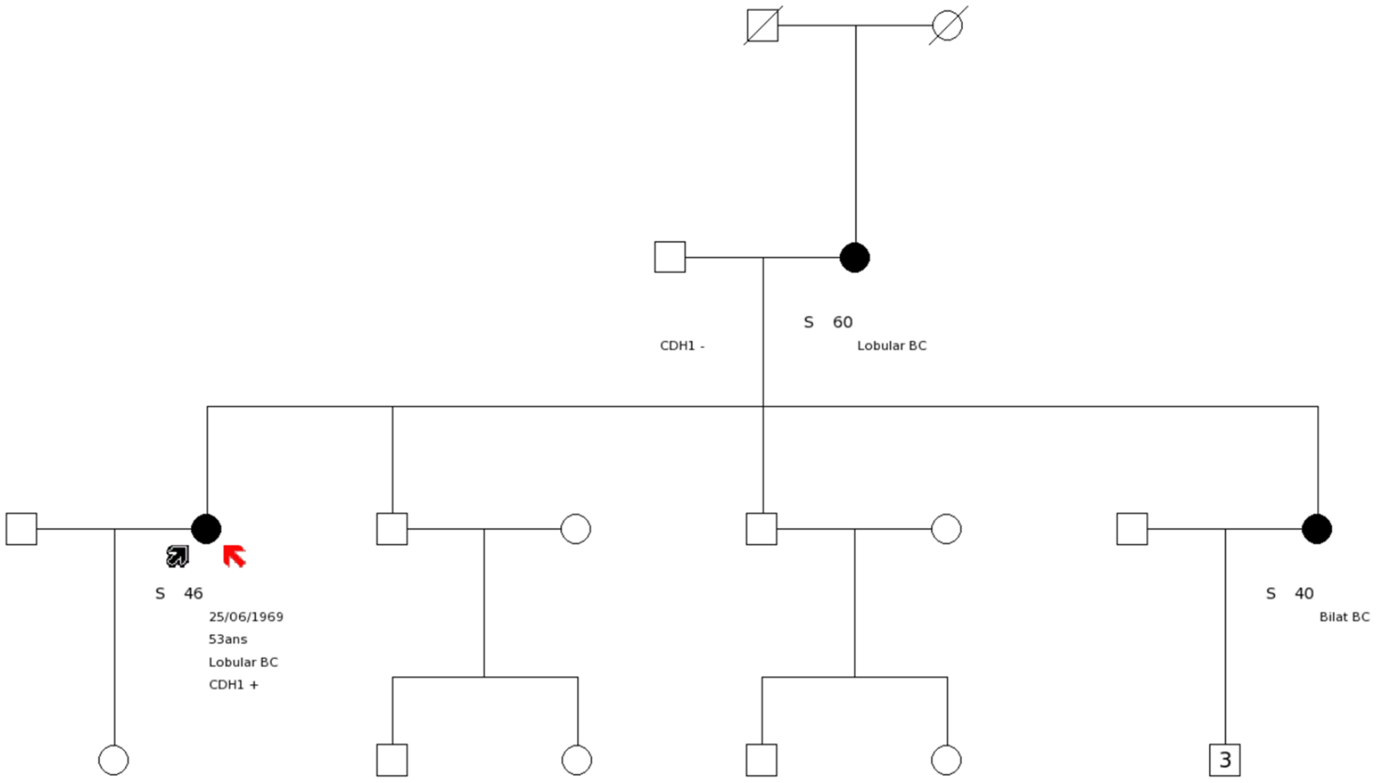

S 60
CDH1 -
Lobular BC
S 46
25/06/1969
53ans
Lobular BC
CDH1 +
S 40
Bilat BC
3

Family 8 :

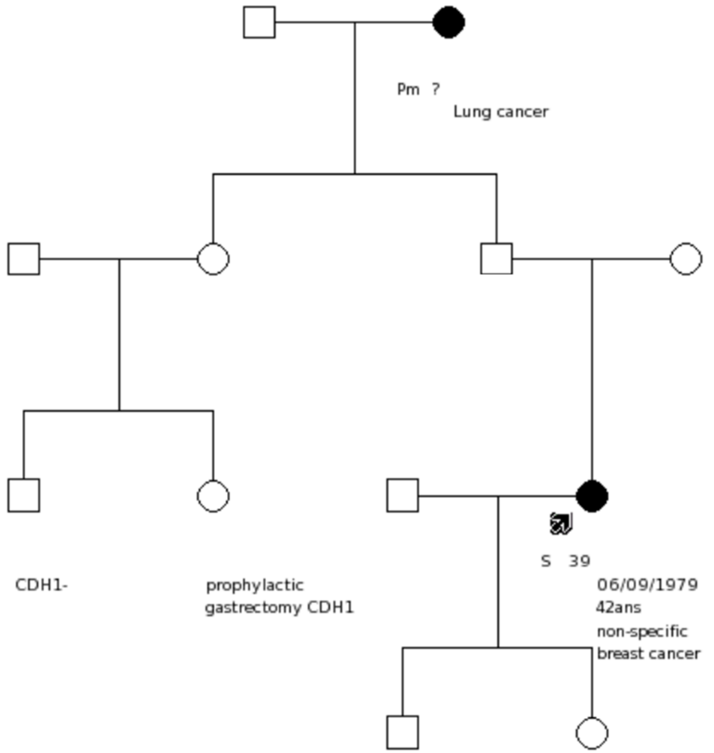
Pm ?
Lung cancer
CDH1-
prophylactic
gastrectomy CDH1
S 39
06/09/1979
42ans
non-specific
breast cancer

Family 9

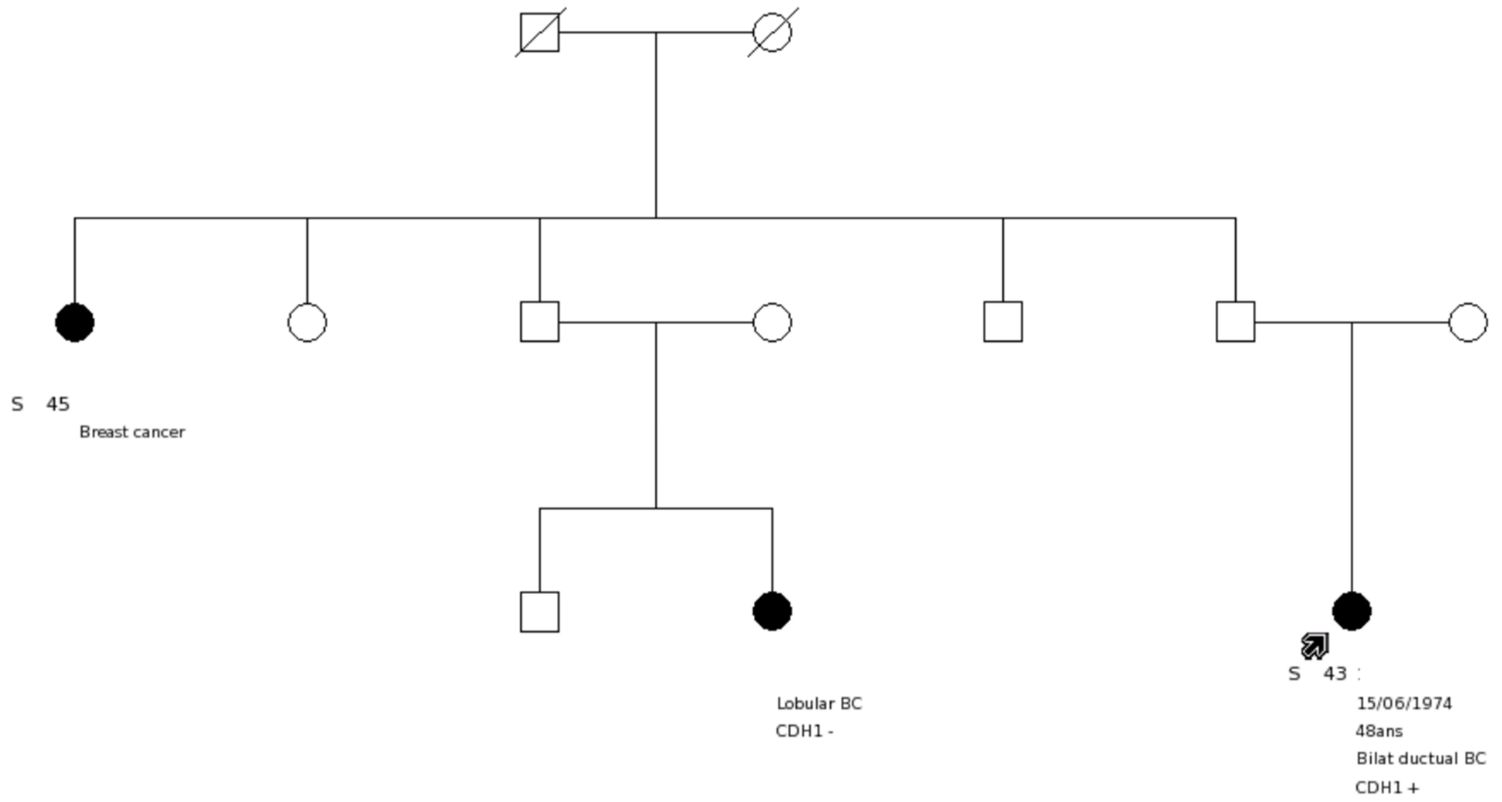
S 45
Breast cancer
Lobular BC
CDH1 -
S 43 :
15/06/1974
48ans
Bilat ductual BC
CDH1 +

Family 10 :

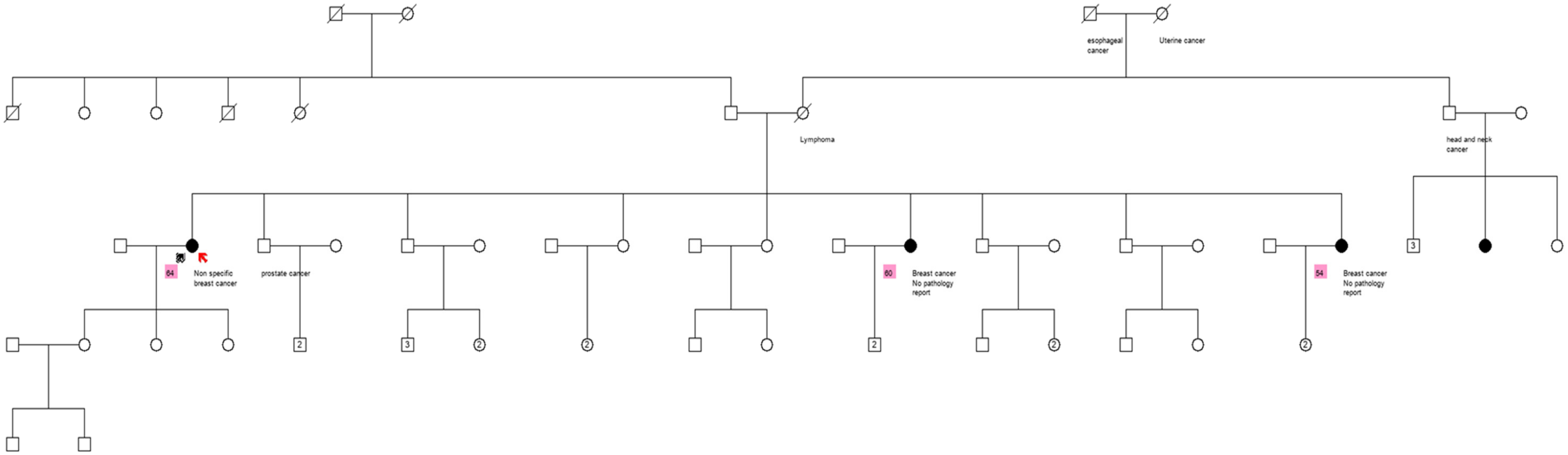

esophageal cancer
Uterine cancer
Lymphoma
head and neck cancer
64
Non specific breast cancer
prostate cancer
60
Breast cancer No pathology report
54
Breast cancer No pathology report

Family 11 :

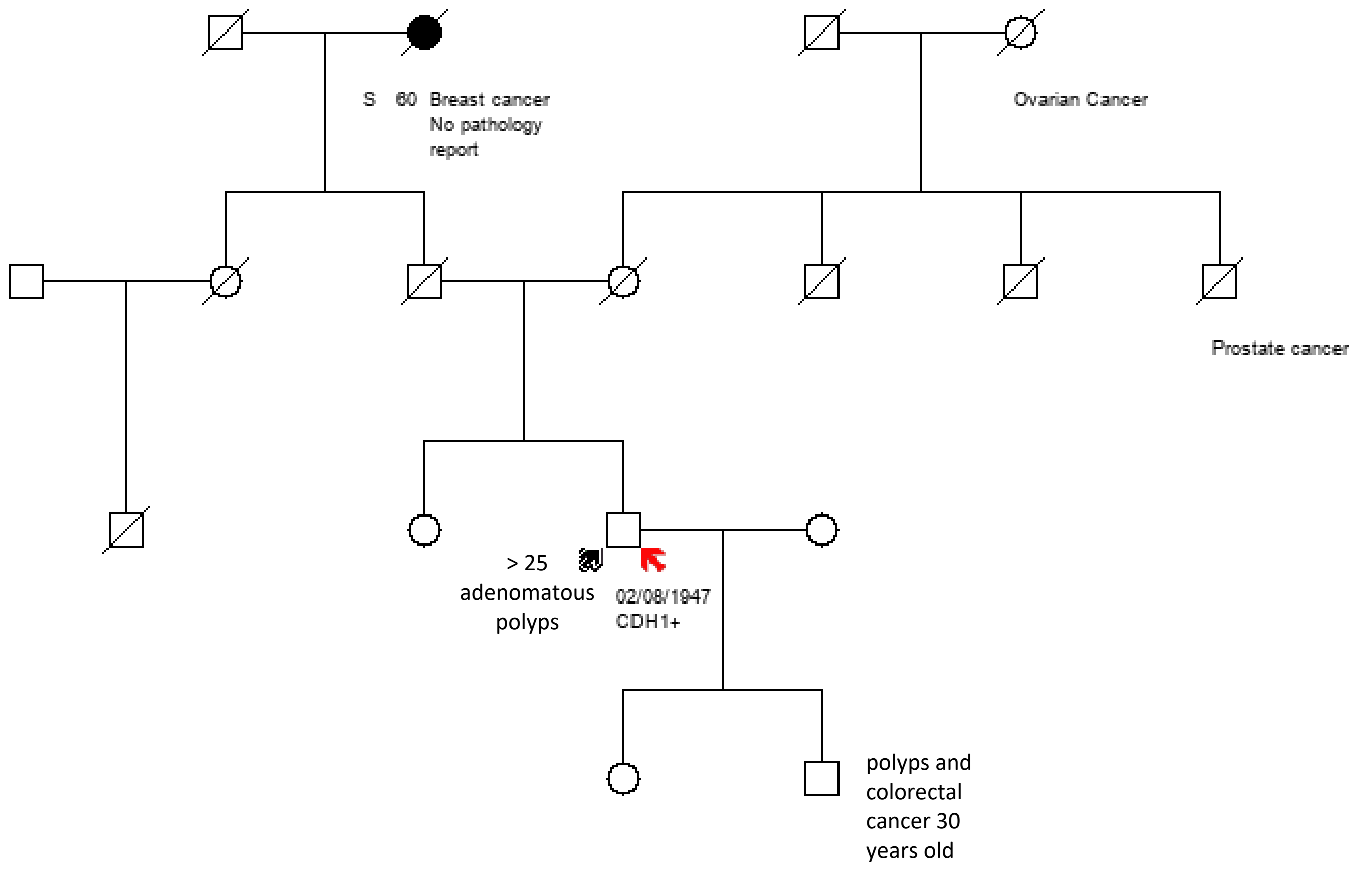
S 60 Breast cancer
No pathology
report
Ovarian Cancer
Prostate cancer
> 25
adenomatous
polyps
02/08/1947
CDH1+
polyps and
colorectal
cancer 30
years old
